# Uptake of a Switching Program for Patients Receiving Intravenous Infliximab and Vedolizumab to Subcutaneous Preparations

**DOI:** 10.3390/jcm11195669

**Published:** 2022-09-26

**Authors:** Gemma Burdge, Alice Hardman, Isabel Carbery, Giacomo Broglio, Dan Greer, Christian P. Selinger

**Affiliations:** 1 Leeds Teaching Hospital NHS Trust, Gastroenterology, Leeds LS7 4SA, UK; 2 IRCCS Fondazione Policlinico San Matteo, Internal Medicine, 27100 Pavia, Italy; 3 Research Institute at St James Hospital, University of Leeds, Leeds LS9 7TF, UK

**Keywords:** inflammatory bowel disease, infliximab, vedolizumab

## Abstract

Background: Recent trials support the clinical efficacy and safety of subcutaneous infliximab (IFX) or vedolizumab (VDZ) for Inflammatory Bowel Disease (IBD). We evaluated the uptake and rationale for choosing to switch from intravenous infusions to subcutaneous injections. Methods: Retrospective analysis of all adult patients receiving standard dosing IFX or VDZ maintenance therapy to investigate uptake of subcutaneous injections and the rationale for switching to subcutaneous injections. Results: Of 232 eligible patients (total = 258: IFX = 190, VDZ = 68, and no longer eligible = 26), 58% of patients on IFX and 59% of patients on VDZ chose to switch to subcutaneous treatment. Age, sex, diagnosis, drug, line of treatment, and duration of treatment were not predictors for willingness to switch. Questionnaire responses (*n* = 51) demonstrate that the decision to switch was not influenced by COVID-19 exposure risk, impact on wider IBD service provision, impact on patient mental health, financial savings, seeking support following a switch, or a sense of independence managing IBD. Switchers (68%) were more motivated by time savings than non-switchers (25%; *p* = 0.0042). Conclusions: Switch uptake rates were 58%, with 90% of patients eligible to switch. Switch decision was influenced by time savings for patients but not by other patient-related factors.

## 1. Introduction

Inflammatory Bowel Diseases (IBD), largely comprising Crohn’s disease (CD) and ulcerative colitis (UC), are chronic inflammatory conditions with significant impacts on a patient’s quality of life [1]. Patients with IBD experiencing suboptimal responses to conventional treatment options often require biological therapy [1,2]. Infliximab (IFX) and vedolizumab (VDZ) treatment is very effective for IBD [3,4,5,6] but has previously been reliant on intravenous (IV) administration of biologic therapies. IV administration can have a bigger impact on patients’ lives due to the time taken to travel to the infusion unit, longer administration time compared to subcutaneous (SC) biologics, and costs associated with travel and/or parking. As in some units biologics are administered by nurses experienced in the management of IBD, patients may feel reassured of the more frequent opportunity for disease assessment and questions when attending for IV therapies. From a provider perspective, however, administration of SC injections could reduce the financial burden and time required to attend infusions and allow IBD service providers to manage increasing clinical demand. Numbers of infusions administered at our IBD unit increased 3-fold between 2015 and 2020, with significant implications, including delays to starting biologic treatment, increased demand for specialist IBD nurses, and restrictions in the provision of clinics and helpline services, which would have otherwise been provided by the IBD nurses. In addition, during the COVID-19 pandemic, some patients may opt for SC therapies in the safety of their home, to avoid travel to infusion units and thus greater exposure to others during their infusion visits.

SC formulations of IFX and VDZ have been developed in recent years and registration trials have demonstrated the clinical efficacy and safety of subcutaneous (SC) biologic treatment for IBD maintenance therapy [7,8]. A recent UK audit, assessing the switching of 163 patients, supports the switch from IV to SC IFX therapy without clinical concern [9]. We initiated an SC switching program for patients on IV IFX or VDZ maintenance therapy. The aim of this study was to evaluate the uptake of the switch offer and rationale for choosing to switch from IV infusions to SC injections in a cohort of IBD patients in west Yorkshire, including the impact of the COVID-19 pandemic on local IBD service provision.

## 2. Materials and Methods

### 2.1. Setting, Patient Cohort, and Switch Process

Leeds Teaching Hospitals provides IBD care to approximately 4000 patients from the local area and tertiary referral patients from the region. IFX and VDZ infusions are administered by an experienced team of IBD nurses in a dedicated IBD infusion unit. All adult patients receiving standard dosing maintenance IV IFX (5 mg/kg bodyweight every 8 weeks) or VDZ (300 mg every 8 weeks) therapy at a tertiary IBD centre in West Yorkshire were offered a switch to the respective SC formulations. All patients with IBD, including those with perianal Crohn’s disease, were approached. We did not restrict the switch offer to patients in clinical remission or response. Exclusion criteria included patients not on vedolizumab or IFX and those on intensified dosing regimes. Patients were informed of the opportunity to switch by a letter (Supplementary Material) outlining the potential benefits of the switch, including potential time and cost savings for the patient, avoidance of unnecessary contact with others during the COVID-19 pandemic, and improvements of the IBD service by redirecting nurses from infusions to other vital parts of the service. Patients were then invited to discuss the switch option with the IBD nurses at the next infusion visit. Those opting to switch were then switched to SC treatment 8 weeks after the last infusion visit. The IBD service aimed for a target of 80% uptake of the switch offer.

### 2.2. Data Collection

Data collection occurred retrospectively between 4 and 5 months after the switch offer. We investigated the uptake of the switch from IV infusions to SC injections by examination of the patient’s electronic health records, extracting data on age, sex, diagnosis, type of IV biologic, line of biological therapy, and treatment duration. All patients were invited to partake in a service evaluation by postal letters. The service evaluation survey was developed by an IBD specialist nurse, a trainee doctor, and a consultant gastroenterologist with subspecialty interest of IBD (see Supplementary Material). We collected data from responders using an anonymous online survey (Google Forms, Google LLC Menlo Park, CA, USA). These included basic patient demographics, patients perception of risk for developing severe COVID-19 illness, costs of travel, time required for travel, satisfaction with the information provided, perception of any pressure to switch, time given to make the decision, perceived SC injection safety and efficacy, potential impact on mental health, importance of impact on wider IBD service provision, and intention to continue with SC/IV treatment in the future. Patients were asked to rate their agreement or disagreement using a 5-point Likert scale. For the analysis, we classified strongly agree and agree as agreement with the statement and neither agree nor disagree, disagree, and strongly disagree as not agreeing with the statement.

### 2.3. Analysis Plan

We used a sample of convenience without a formal sample size calculation due to the nature of the study. Categorical data were presented as percentages and compared using chi-square tests. Continuous data were presented as medians and compared by Kruskal–Wallis tests. All analyses were performed using SPSS version 26 (IBM Corp. Released 2019. IBM SPSS Statistics for Windows, Version 26.0. Armonk, NY, USA: IBM Corp).

## 3. Results

### 3.1. Acceptance of Switch Option and Factors Associated with Switch

Of the 258 identified patients (190 IFX, 68 VDZ), 26 (21 IFX, 5 VDZ) were no longer eligible to switch therapy as they had been changed to an intensified treatment schedule no longer eligible for switch (6), been switched to a different biologic (12), moved out of area (2), ceased treatment for clinical reasons (5), or required surgery (1). Of the remaining 232 patients, 135 switched to SC (98 (58%) IFX, 37 (59%) VDZ). The remaining 97 declined a switch to SC (71 IFX, 26 VDZ). There were no significant predictors for willingness to switch relating to patient age, sex, diagnosis, drug, line of treatment, or duration of treatment (Table 1, *p* > 0.05).

### 3.2. Questionnaire Responses

**Figure 1:**
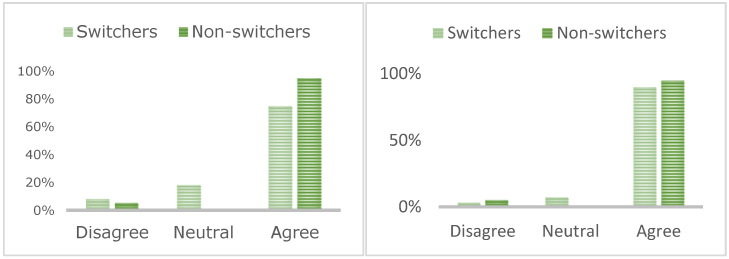
Patients who value face-to-face contact at the IBD unit (**left**) and those who utilise this time to seek support for management of their IBD (**right**).

Patient questionnaire responses (*n* = 51, 22%) demonstrated that a decision to switch was not influenced by COVID-19 exposure risk, the impact on wider IBD service provision, impact on patient mental health, financial savings, seeking support following a switch, or a sense of independence managing IBD. Switchers (68%) were more motivated by time savings than non-switchers (24%; Table 2; *p* = 0.0042). Most patients valued face-to-face support at the IBD unit (90% switchers, 96% non-switchers; Figure 1) and utilised this time to seek support for managing IBD (75% switchers, 95% non-switchers).

**Figure 2:**
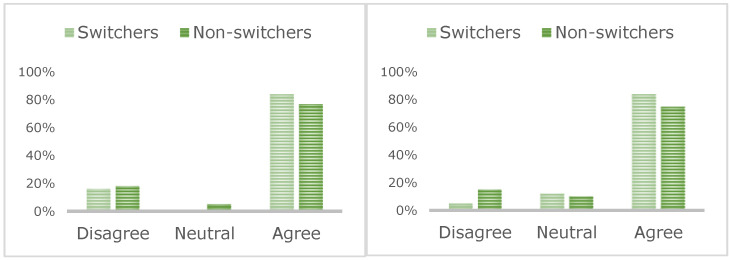
Patient consensus about enough information (**left**) and time (**right**) being given during the switching process.

Most patients felt they received enough time (84% switchers, 76% non-switchers) and information (84% switchers, 77% non-switchers; Figure 2) to make a decision about switching. In total, 61% of switchers reported concerns about the efficacy of SC injections compared to only 28% of non-switchers. Most patients reported being happy with their decision (80% switchers, 76% non-switchers), although 31% of non-switchers were considering a switch.

Differences in employment status between switchers (84% employed) and non-switchers (58% employed) were not statistically significant (*p* = 0.0876).

A total of 86% of patients travelled by car, 4% by public transport, 6% by taxi, and 4% walked to the infusion unit prior to switching. They paid GBP 4 on average for car parking during their visit. Mode of transport to the infusion unit was not associated with willingness to switch (Table 3; *p* = 1). There were no differences in time spent for the infusion visit (includes travel and infusion time), with switchers reporting 147 min (median) versus non-switchers reporting 137 min (*p* = 0.63).

## 4. Discussion

The advent of SC formulations of IFX and VDZ offers patients and IBD units the opportunity to switch patients established on IBD therapy while being maintained on the same biologic [7,8]. Our study is the first to report the uptake of a switch offer by patients in a large cohort and the factors associated with willingness to switch. We report switch uptake rates of 58% and found no evidence that a decision to switch was influenced by age, sex, diagnosis, drug, line of treatment, or duration of treatment. Time saved by not attending an IV infusion was significantly associated with willingness to switch (*p* = 0.0042).

Switching to SC formulations may have several potential advantages for patients and the IBD service. First, the patient may save considerable time, as our patients reported on average spending 140 min for travel and attendance per IV infusion. This is contrasted by four SC injections taking approximately 10 min each for the same 8-week treatment cycle. This was clearly appreciated by patients opting for the switch. Second, patients may also save money from transport and parking, but we did not find that our patients placed much focus on cost when deciding whether to switch to SC. Third, the risk of potential exposure to COVID-19 while attending an infusion has been a concern early in the pandemic. Reducing the risk of this exposure was a key recommendation early on in 2020, and the switches in Kettering and Liverpool were largely motivated by this [9,10]. In our cohort, this did not seem to be an important motivator for switching. This may relate to the fact that we offered the switch in June 2021 when the UK COVID vaccine program had already been rolled out for all patients on IFX and VDZ [11]. Our infusion unit is already based at the non-acute hospital side, which may be perceived to be lower risk than a hospital site treating COVID-19 patients on the same premises. Fourth, by reducing the number of infusions delivered by IBD specialist nurses, these expert nurses could be offering more clinics for IBD patients, more visits to IBD inpatients, and speedier response to the IBD hotline for flares. These aspects of the IBD service had come under severe strain recently and we were surprised to find the this did not seem to influence our patients’ decision making regarding SC switch.

Few studies have previously reported on patient preference comparing IV versus SC formulations [12]. Allen et al. examined patient choice when offered IV IFX versus SC adalimumab and found only a non-significant preference for IFX [12]. Switching IV IFX to SC adalimumab is not encouraged, as this has been associated with a greater loss in response rate [13]. Switching from SC to IV with the same molecules is, however, safe and effective [7,8,9]. Previous work from our group found that in patients already receiving biological therapy, the acceptance of both IV and SC administration is good [14].

The implementation of the switch program with initial approach by letter followed by the opportunity to discuss the switch option with an IBD nurse at the next infusion appointment was appreciated by our patients, as the vast majority of patients expressed that they were provided with sufficient information and that they felt no pressure to switch. The main motivation for switching seemed to be how much patients valued saving time. There was no difference in time spent for infusions between switchers and non-switchers but the potential for time saving did not motivate non-switchers. Interestingly, cost aspects and COVID-19 risk did not seem to influence decision making. Importantly, 31% of non-switchers stated that they now are open to the idea of switching. This offers the opportunity to increase the switch uptake and come closer to our self-set target of 80% switch uptake.

In addition to the reduced demand for infusions within the IBD infusion unit, a switch to SC formulations may also affect the IBD service financially, depending on the infusion unit cost and medication cost differences between the IV and SC formulations. This may be an additional motivator for clinicians to initiate switches. We did not include cost aspects in our switch invitation letter (Supplementary Material). Some clinicians may be concerned about non-adherence with SC therapies or potential disease flares after switching. While we did not examine this in our study, they should feel reassured by the observational clinical outcomes published in observational real-world studies and the registration clinical trial [7,8,9].

The main strength of this study is the uniform approach to the switch process, with a clear focus on patient choice and the absence of perceived pressure to switch. We had access to full clinical records and therefore completeness of outcomes for the switch decision. There are a number of limitations. This is a single-centre experience and the local circumstances may not apply in other centres. Our survey response rate was *n* = 55 (22%), which is in line with expectations; however, there is an obvious risk of response bias. Switch rates may also be higher now than when we collected our data in October 2021.

## 5. Conclusions

We have established that 58% of patients agreed to switching IV IFX and VDZ to SC formulations when initially offered the switch. Patients willing to switch seemed to be motivated by saving time otherwise spent on travel and administration of IV infusions. 

## Figures and Tables

**Table 1:** Cohort characteristics and factors potentially associated with willingness to switch.

Criteria	Switch	Non-Switch	*p*-Value
Age (mean years + IQR)	41.5+/−14.0	43.2+/−16.7	*p* = 0.42
Sex	Male 60 (44.4%)	Male 52 (53.6%)	*p* = 0.18
	Female 75 (55.6%)	Female 45 (46.4%)	
Diagnosis	CD 70 (51.9%)	CD 47 (48.4%)	*p* = 0.65
	UC 49 (36.3%)	UC 37 (38.1%)	
	IBDU 13 (9.6%)	IBDU 13 (13.4%)	
Line of treatment	1st 94 (69.6%)	1st 63 (64.9%)	*p* = 0.52
	2nd 27 (20%)	2nd 23 (23.7%)	
	3rd 12 (8.9%)	3rd 7 (7.2%)	
	4th 2 (1.5%)	4th 4 (4.1%)	
Drug	IFX 98 (72.6%)	IFX 71 (73.2%)	*p* = 1.0
	VDZ 37 (27.4%)	VDZ 26 (26.8%)	
Duration of treatment (years)	4.3+/−3.4	3.9+/−3.4	*p* = 0.44

Crohn’s disease (CD), ulcerative colitis (UC), Inflammatory Bowel disease-Unclassified (IBDU), Infliximab (IFX), Vedolizumab (VDZ)

**Table 2:** Patient attitudes towards SC switch and satisfaction with the decision made.

	Switch (31)	Non-Switch (20)	*p*-Value
Sufficient information provided	25/31	15/20	*p* = 0.73
Felt pressure to switch	2/31	2/20	*p* = 0.64
Patient concerns efficacy of SC	14/31	5/20	*p* = 0.24
Patient concerns safety of SC	7/31	2/20	*P* = 0.45
COVID risk influenced decision	7/31	2/20	*p* = 0.45
Time saving influenced decision	21/31	5/20	*p* = 0.0042
Cost saving by patient influenced decision	9/31	5/20	*p* = 1
Potential impact of switch on mental health influenced decision	9/31	9/20	*p* = 0.37
Impact of infusion service on IBD service influenced decision	17/31	8/20	*p* = 0.39
I value the support of face to face in infusion unit	24/31	19/20	*p* = 0.13
Happy with decision	24/31	16/20	*p* = 1

**Table 3:** Mode of transport used prior to invitation to switch.

	Switchers	Non-Switchers
Car	27	16
Public transport	1	1
Taxi	1	2
Walk/bicycle	2	0

## Data Availability

Summary data are available on request.

## Supplementary Materials

The following supporting information can be downloaded at: https://www.mdpi.com/article/10.3390/jcm11195669/s1, Letter: IBD treatment service evaluation (SCSW) (live).
