# Supplementary material for: Uptake of a Switching Program for Patients Receiving Intravenous Infliximab and Vedolizumab to Subcutaneous Preparations
Source: J Clin Med. 2022 Sep 26;11(19):5669. doi: 10.3390/jcm11195669 (PMC9571673; doi:10.3390/jcm11195669)
Supplement: Supplementary file 1 [file jcm-11-05669-s001.zip › jcm-1870682-supplementary.pdf]

Leeds Inflammatory Bowel Disease Unit

Patient address

Dear ______________

We are writing to you to propose a change in the way of giving your Infliximab treatment. There are now two formulations of Infliximab available for treatment of your inflammatory bowel disease (IBD). In addition to the intravenous infusion which is given to you at our IBD infusion unit, there is now a subcutaneous injection which can be self-administered at home. We recommend a switch to the subcutaneous injection you can administer yourself.

There are a number of reasons why we think a switch to the subcutaneous injection is a good idea. We believe this will benefit you directly and indirectly as well as your fellow IBD patients.

1. You won't have to attend for an infusion thereby reducing the time you need to spend on your IBD care. This might suit a busy lifestyle better as you will only need to plan for 10 minutes for your injection rather than spending 60-90 minutes at the infusion unit.
2. You will be able to avoid the travel to the infusion unit saving on travel costs and parking fees as well as your time.
3. You will be able to reduce your risk of being exposed to COVID (or in future other potential infectious diseases) by avoiding the busy infusion unit and travel. By administering the treatment at home you can in these critical times reduce your risk.
4. The IBD nurses are currently overstretched and spend a lot of time delivering infusions. Unfortunately other aspects of the service like the helpline, visiting inpatients, attending clinic and providing nurse led clinics are currently suffering because of this. By switching patients to the subcutaneous injection we can free up the IBD nurses to improve timeliness of helpline responses, restart visiting IBD patients when they are admitted to hospital, providing nurse support in consultant clinics and reduce waiting times for outpatient appointments by providing extra clinic capacity.

We will speak to you about the proposed switch when you come for your next infusion. Only those patients that agree will be switched to the subcutaneous injection. We are happy to answer all your questions when you come for your next infusion. Below you will find our frequently asked questions that may address some of your questions in the meantime.

Why is the change being made?
The company that makes Infliximab recognises that it may be difficult or inconvenient for patients to attend the infusion unit regularly to have their infusions. They have created a subcutaneous formulation that can be administered at home.

What formulations of Infliximab are available?
There is a vial that is administered by our nurses in the IBD infusion unit by intravenous infusion. This means the medication is delivered directly into the circulation through a vein, usually in your arm. There is also a pre-filled pen or pre-filled syringe which is given as an injection under the skin (subcutaneous injection).

Will subcutaneous injections be just as safe and effective as intravenous infusions?
We appreciate the concerns that some of our IBD patients have about switching to a new formulation of Infliximab, such as whether a different formulation will still be effective and about having less face-to-face contact with our IBD nursing team. The European Medicines Agency (EMA) has approved the safety of this formulation and the company has demonstrated in large clinical trials that the two formulations are equally safe and effective. We will also monitor you carefully if you change formulation and will arrange a telephone appointment with our IBD team 6 months after swapping formulation. In addition colleagues in Liverpool have switched 132 patients to subcutaneous Infliximab with excellent efficacy and safety results.

Am I likely to experience side effects after changing?
You are no more likely to experience side effects on subcutaneous Infliximab than you would with intravenous Infliximab. You may experience a small reaction at the injection site. We will continue to monitor you closely and would like you to let us know if you experience any side effects.

When is the change being made?
We will now be offering the choice of formulation. We will also ask at your next infusion if you want to switch.

Is subcutaneous formulation a suitable treatment for me?
We have identified you as patient that is suitable for the switch.

How will I receive the subcutaneous injections?
If you choose to have subcutaneous injections, a supply of injections will be delivered to your home regularly by an external homecare company.

Will I be shown how to use the subcutaneous injections?
A nurse will visit your home to show you how to use the pre-filled pen or syringe. They will visit 2 or 3 times at which point you should feel comfortable to inject yourself.

How often will I need to inject?
You will need to inject yourself every two weeks. If you are currently receiving Infliximab regularly, your first subcutaneous injection should be administered 8 weeks after your last intravenous infusion.

I would like to discuss the change, how can I do this?
If you would like to discuss the change, you can speak to us at your next infusion. Alternatively you can email leedsth-tr.ibd@nhs.net.

IBD treatment service evaluation (SCSW) (live)

Page 1: Page 1

This questionnaire aims to gather information to understand why patients have chosen to switch to self-administered subcutaneous Infliximab or Vedolizumab injections, assess the impact of IBD treatment delivery on patient quality of life, and identify areas where our service can be improved. Your responses are kept completely anonymous. Thank you for your feedback.

1. What is your age group? ✱ *Required*

- ○ Under 18
- ○ 18-30
- ○ 31-45
- ○ 46-60
- ○ 61-75
- ○ Over 75

2. What is your education or employment status? (please select all that apply) ✱ *Required*

- ☐ Part-time education
- ☐ Full-time education
- ☐ Part-time employment

- [ ] Full-time employment
- [ ] Full-time parent
- [ ] Not currently employed
- [ ] Retired
- [ ] Other

2.a. If you selected other please specify:

____

3. In terms of developing serious illness from Covid-19, I consider myself to be: ✱ *Required*

- ( ) Low risk
- ( ) Medium risk
- ( ) High risk

4. How did you previously travel to attend your hospital infusion appointments? (Please select all that apply) ✱ *Required*

- [ ] Private motorised vehicle (car, motorbike)
- [ ] Taxi
- [ ] Public transport (bus, train)
- [ ] Bicycle
- [ ] Walk

5. On average, how long did it take to travel for a hospital infusion (round trip including

transport each way, duration of infusion, observation period)? ✱ *Required*

6. If you drove a private motorised vehicle, what was the distance of the journey (in miles, one-way?) ✱ *Required*

7. How much did you spend on parking costs for each infusion visit (if applicable)?

8. How much did you spend on public transport for each infusion visit (if applicable)?

9. To what extent do you agree with the following statements about the switching process:

	To what extent do you agree with the following statements about the switching process:				
	Strongly disagree	disagree	neither agree nor disagree	agree	strongly agree

I received enough information about the pros and cons of switching	○	○	○	○	○
I was given enough time to make a decision about switching	○	○	○	○	○
I felt pressured to make the switch to home injections	○	○	○	○	○

10. To what extent did the following factors concern you when making a decision about switching:

	To what extent did the following factors concern you when making a decision about switching:				
	Strongly disagree	disagree	neither agree nor disagree	agree	strongly agree
The effectiveness of subcutaneous injections	☐	☐	☐	☐	☐
The safety of subcutaneous injections	☐	☐	☐	☐	☐
Seeking support for my IBD following the switch	☐	☐	☐	☐	☐
Risk of exposure to Covid-19	☐	☐	☐	☐	☐

11. To what extent did the following factors concern you when making a decision about switching:

	To what extent did the following factors concern you when making a decision about switching:				
	Strongly disagree	disagree	neither agree nor disagree	agree	strongly agree

Saving time	☐	☐	☐	☐	☐
Saving money e.g. travel costs	☐	☐	☐	☐	☐
The impact on my mental health	☐	☐	☐	☐	☐
Feeling a sense of independence when managing my IBD	☐	☐	☐	☐	☐
The wider impact on IBD service provision	☐	☐	☐	☐	☐

12. To what extent do you agree with the following statements about receiving intravenous injections at the IBD unit:

	To what extent do you agree with the following statements about receiving intravenous injections at the IBD unit:				
	Strongly disagree	disagree	neither agree nor disagree	agree	strongly agree
It was convenient for my lifestyle	☐	☐	☐	☐	☐
I valued the face to face support at the IBD unit	☐	☐	☐	☐	☐
I utilised visits to the day centre to address other questions and concerns about my IBD	☐	☐	☐	☐	☐

13. To what extent do you agree with the following statements about the future of your IBD treatment:

	To what extent do you agree with the following statements about the future of your IBD treatment:				
	Strongly disagree	disagree	neither agree nor disagree	agree	strongly agree
I am happy I switched to self-administered subcutaneous injections	☐	☐	☐	☐	☐
I am happy to self-administer subcutaneous injections in the short term	☐	☐	☐	☐	☐
I am happy to self-administer subcutaneous injections in the long term	☐	☐	☐	☐	☐
I would like to switch back to intravenous infusions at some point in the future	☐	☐	☐	☐	☐

14. Were there any other factors (either motivations or concerns) which affected your decision when choosing whether to switch to self-administered injections? Please give as much detail as possible

15. Are there any aspects of the switching process which you feel could be improved? Please give as much detail as possible

Page 2: Final page

Thank you very much for taking part in this survey.

The Leeds IBD service

IBD treatment service evaluation (NSW) (live)

Page 1: Page 1

This questionnaire aims to gather information to understand why patients have chosen to continue to attend for intravenous Infliximab or Vedolizumab infusions at our IBD infusion unit, assess the impact of IBD treatment delivery on patient quality of life, and identify areas where our service can be improved. Your responses are kept completely anonymous. Thank you for your feedback.

1. What is your age group? ✱ *Required*

- ◯ Under 18
- ◯ 18-30
- ◯ 31-45
- ◯ 46-60
- ◯ 61-75
- ◯ Over 75

2. 2. What is your education/employment status? (Please select all that apply) ✱ *Required*

- ☐ Part-time education
- ☐ Full-time education
- ☐ Part-time employment

- [ ] Full-time employment
- [ ] Full-time parent
- [ ] Not currently employed
- [ ] Retired
- [ ] Other

2.a. If you selected Other, please specify:

[ ]

3. In terms of developing serious illness from Covid-19, I consider myself to be: ✱ *Required*

- ○ Low risk
- ○ Medium risk
- ○ High risk

4. How did you previously travel to attend your hospital infusion appointments? (Please select all that apply) ✱ *Required*

- [ ] Private motorised vehicle (car, motorbike)
- [ ] Taxi
- [ ] Public transport (bus, train)
- [ ] Bicycle
- [ ] Walk

5. On average, how long did it take to travel for a hospital infusion (round trip including

transport each way, duration of infusion, observation period)? ✱ *Required*

6. If you drove a private motorised vehicle, what was the distance of the journey (in miles, one-way?) ✱ *Required*

7. How much did you spend on parking costs for each infusion visit (if applicable)?

8. How much did you spend on public transport for each infusion visit (if applicable)?

9. To what extent do you agree with the following statements about the switching process:

	To what extent do you agree with the following statements about the switching process:				
	Strongly disagree	disagree	neither agree nor disagree	agree	strongly agree

I received enough information about the pros and cons of switching	○	○	○	○	○
I was given enough time to make a decision about switching	○	○	○	○	○
I felt pressured to make the switch to home injections	○	○	○	○	○

10. To what extent did the following factors concern you when making a decision about switching:

	To what extent did the following factors concern you when making a decision about switching:				
	Strongly disagree	disagree	neither agree nor disagree	agree	strongly agree
The effectiveness of subcutaneous injections	☐	☐	☐	☐	☐
The safety of subcutaneous injections	☐	☐	☐	☐	☐
Seeking support for my IBD following the switch	☐	☐	☐	☐	☐
Risk of exposure to Covid-19	☐	☐	☐	☐	☐

11. To what extent did the following factors concern you when making a decision about switching:

	To what extent did the following factors concern you when making a decision about switching:				
	Strongly disagree	disagree	neither agree nor disagree	agree	strongly agree

Saving time	☐	☐	☐	☐	☐
Saving money e.g. travel costs	☐	☐	☐	☐	☐
The impact on my mental health	☐	☐	☐	☐	☐
Feeling a sense of independence when managing my IBD	☐	☐	☐	☐	☐
The wider impact on IBD service provision	☐	☐	☐	☐	☐

12. To what extent do you agree with the following statements about receiving intravenous injections at the IBD unit:

	To what extent do you agree with the following statements about receiving intravenous injections at the IBD unit:				
	Strongly disagree	disagree	neither agree nor disagree	agree	strongly agree
It was convenient for my lifestyle	☐	☐	☐	☐	☐
I valued the face to face support at the IBD unit	☐	☐	☐	☐	☐
I utilised visits to the day centre to address other questions and concerns about my IBD	☐	☐	☐	☐	☐

13. To what extent do you agree with the following statements about the future of your IBD treatment:

	To what extent do you agree with the following statements about the future of your IBD treatment:				
	Strongly disagree	disagree	neither agree nor disagree	agree	strongly agree
I am happy I chose not to switch to self-administered injections	☐	☐	☐	☐	☐
I am happy to receive intravenous infusions in the short term	☐	☐	☐	☐	☐
I am happy to receive intravenous infusions in the long term	☐	☐	☐	☐	☐
I would like to switch to self-administered injections at some point in the future	☐	☐	☐	☐	☐

14. Were there any other factors (either motivations or concerns) which affected your decision when choosing whether to switch to self-administered injections? Please give as much detail as possible

15. Are there any aspects of the switching process which you feel could be improved? Please give as much detail as possible

Page 2: Final page

Thank you very much for taking part in this survey.

The Leeds IBD service
